# The complete chloroplast genome sequence of *Dendrobium wangliangii* (Orchidaceae)

**DOI:** 10.1080/23802359.2020.1827063

**Published:** 2020-10-07

**Authors:** Shi-Cheng Shao, Lu Tang, Yan Luo

**Affiliations:** a Gardening and Horticulture Department, Xishuangbanna Tropical Botanical Garden, Chinese Academy of Sciences, Yunnan, P.R. China; b College of Forestry, Shanxi Agricultural University, Shanxi, P.R. China; c Gardening and Horticulture Department, Core Botanical Gardens, Chinese Academy of Sciences, Yunnan, P.R. China

**Keywords:** *Dendrobium wangliangii*, chloroplast genome, phylogenetic analysis Orchidaceae

## Abstract

*Dendrobium wangliangii* is a rare orchid species with extremely small populations, endemic to China. In this study, the complete chloroplast (cp) genome sequence and the genome features of *D. wangliangii* were analyzed. The whole cp genome sequence of *D. wangliangii* is 160,052 including a large single-copy region (LSC, 87,525 bp), a small single-copy region (SSC, 18,373 bp), and a pair of repeat regions (IRs, 27,077 bp, each). The contents of four bases in cpDNA were A (30.9%), C (18.9%), G (18.3%) and T (31.9%), respectively. The total content of GC is 37.1%. The cp genome contains 129 genes, consisting of 124 unique genes (78 protein-coding genes, 38 tRNAs, and 8 rRNAs). Phylogenetic analysis showed that *D. wangliangii* nested with other *Dendrobium* spp. and was closely related to *D. ellipsophyllum, D. wattii* and *D. longicornu*.

*Dendrobium* is one of the largest genera in the orchid family. It consists of approximately 1500 species, mainly distributed in tropical and subtropical regions of the world (Pridgeon et al. 2014). Seventy-eight species of this genus in 14 sections were recorded in China (Zhu et al. 2009). However, molecular phylogenies of the combination of partial chloroplast marks and ITS-rDNA are not always consistent to morphological sections (Xiang et al. 2016). Additional molecular marks such as complete chloroplast genome sequences are needed for phylogenetic and evolutionary analyses.

*Dendrobium wangliangii* was discovered as a new species in section *Dendrobium* in 2008 (Hu et al. 2008). *Dendrobium wangliangii* is distributed in specific habitats with narrow distributions, e.g. the Jinshajiang dry-hot valley in Luquan County, Kunming, Yunnan Province (Wang et al. 2019), and the populations are extremely small. Given the special features of morphology and the nature of drought tolerance, phylogenetic position of *D. wangliangii* is unknown. In this study, a complete chloroplast genome (cp genome) sequence of *D. wangliangii* was characterized and analyzed for its phylogenetic position.

The leave samples of *D. wangliangii* was collected in Fumin County, Yunnan, China (25°20′19″N, 102° 27′26″E). Total genomic DNA from leaf was extracted using Tiangen DNA kit (TIANGEN, China), and sequenced by the Illumina Hiseq 2500 sequencing platform (Illumina, CA, USA) at Personal Biotechnology Co., Ltd (Shanghai, China). The DNA of *D. wangliangii* sample was preserved in Xishuangbanna Tropical Botanical Garden, Chinese Academy of Sciences with collection number JSJSH17122018-1. The sequence data were *de novo* assembled to a complete and circular cp genome using GetOrganelle (Jin et al. 2020). Gene annotation was accomplished using GeSeq with default sets (Tillich et al. 2017), and manually checked in Geneious Primer 2020 (Kearse et al. 2012). Sequences of Coding regions (CDS) and rRNAs were extracted, aligned and concatenated in Geneious Primer 2020.

The whole cp genome sequence of *D. wangliangii* (GenBank accession no. MT798588) is totally 160,052 bp in length, in which the contents of four bases in cpDNA were A (30.9%), C (18.9%), G (18.3%) and T (31.9%), respectively. The total content of GC is 37.1%. The structure of cp genome includes a large single-copy region (LSC, 87,525 bp), a small single-copy region (SSC, 18,373 bp), and a pair of inverted repeat regions (IRs, 27,077 bp, each). Typical code ATG was used as the initiator codon and typical code TAA as the terminator codon in all the 78 protein-coding genes. The gene order and features of the *D. wangliangii* are consistent with congener species in *Dendrobium* (Niu, Xue et al. 2017; Niu, Zhu, et al. 2017).

To further investigate its phylogenetic position, the complete chloroplast genomes from 22 species of subfamily Epidendroideae, e.g. 16 *Dendrobium* species, three *Bulbophyllum* species and three *Cymbidium* species, and two species of *Paphiopedilum* from subfamily Cypripedioideae as outgroup, were selected to reconstruct the phylogenetic tree. The best-fit model GTR + F+R2 using the Akaike information criterion (AIC) was applied to read the best model to construct maximum likelihood (ML) trees using IQ-TREE 1.6 (Lam-Tung et al. 2015). Phylogenetic analysis showed that *D. wangliangii* nested in *Dendrobium* clade (Figure 1) and was closely related to *D. ellipsophyllum*, *D. wattii* and *D. longicornu*. This newly reported chloroplast genome of *D. wangliangii* provides important genomic information for *Dendrobium* species phylogenetic and evolutionary analyses.

**Figure 1.**
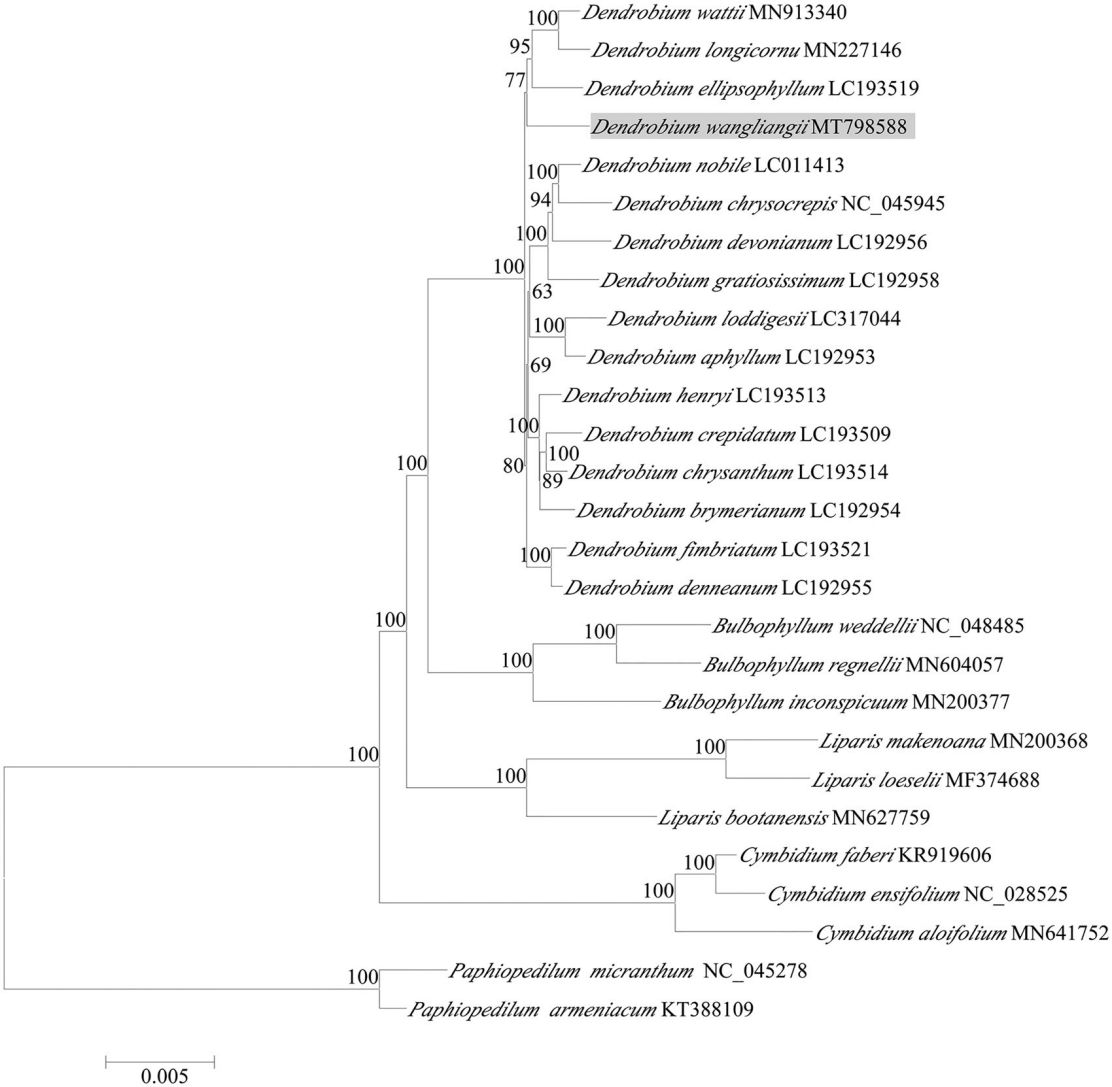
Phylogenetic analysis of *Dendrobium wangliangii* using maximum likelihood of 69 protein-coding genes and four rRNA genes from 16 species from the genus *Dendrobium*, two species of *Paphiopedilum* as outgroup. The support values were indicated on the branch node or right side of node; and each tip was labeled by species name and GenBank accession no. The phylogenetic position of the species *D. wangliangii* was shown in gray box.

## Data Availability

The data that support the analyses and results of *Dendrobium wangliangii* in this paper are available in GenBank with accession no. MT798588 (https://www.ncbi.nlm.nih.gov/). Raw sequencing reads was deposited in SRA with BioProject accession: PRJNA659161. (https://www.ncbi.nlm.nih.gov/Traces/study/?acc=PRJNA659161).
